# Assessing the reliability and validity of the Danish version of Organizational Readiness for Implementing Change (ORIC)

**DOI:** 10.1186/s13012-018-0769-y

**Published:** 2018-06-05

**Authors:** Marie Höjriis Storkholm, Pamela Mazzocato, Mesfin Kassaye Tessma, Carl Savage

**Affiliations:** 1 Department of Learning, Informatics, Management and Ethics, Medical Management Centre, Karolinska Institutet, Tomtebodavägen 18A, 171 77 Stockholm, Sweden; 2 Department of Obstetrics and Gynecology, Aarhus University Hospital, Aarhus, Denmark; 3 Department of Learning, Informatics, Management and Ethics, Karolinska Institutet, Stockholm, Sweden

**Keywords:** Organizational readiness for change, Validation study, Translation, Change management, Implementation, Questionnaire

## Abstract

**Background:**

Organizational change initiatives in health care frequently achieve only partial implementation success. Understanding an organizational readiness for change (ORC) may be a way to develop more effective and efficient change strategies. Denmark, like many countries, has begun a major system-wide structural reform which involves considerable changes in service delivery. Due to the lack of a validated Danish instrument, we aimed to translate and validate a Danish version of the Organizational Readiness for Implementing Change (ORIC) questionnaire. It measures if organizational members are confident in their collective *commitment* towards and ability (*efficacy*) to implement organizational change. ORIC is concise, grounded in theory, and designed, but not yet validated among employees in a real hospital setting.

**Methods:**

The 12-item ORIC instrument was translated into Danish and back-translated to English. Employees (*N* = 284) at a hospital department facing a major organizational change in the Central Denmark Region completed the questionnaire. Face and content validity was ascertained. Exploratory factor analysis (EFA) and a confirmatory factor analysis (CFA) were used to assess construct validity. Reliability was assessed with Cronbach’s alpha. Item response theory (Rasch analysis) was used to determine item and person reliability.

**Results:**

Response rate was 72%. A two factor (commitment and efficacy), 11-item scale, of the Danish language ORIC was shown to be valid (CFI = .95, RMSEA = .067, and CMNI/DF = 2.32) and reliable (Cronbach’s alpha 0.88) in a health care setting. Item response analysis confirmed acceptable person and item separation reliability.

**Conclusions:**

Our version of ORIC showed acceptable validity and reliability as an instrument for measuring readiness for implementing organizational change in a Danish-speaking health care population. For health care managers interested in evaluating their organizations and tailor change strategies, ORIC’s brevity and theoretical underpinnings could make it an appealing and feasible tool to develop more successful change efforts.

**Electronic supplementary material:**

The online version of this article (10.1186/s13012-018-0769-y) contains supplementary material, which is available to authorized users.

## Background

The optimism that signals the start of so many change efforts in health care seems somewhat misguided when decades of research suggest that organizational change initiatives frequently achieve only partial implementation success [1, 2]. Understanding the degree to which organizational members and organizations are “ready” to implement a specific change has been suggested as a way forward. Multiple definitions and multiple instruments to measure organizational readiness for change exist [3]; however, there is no gold standard. Recently, a focus on the supra-individual level has emerged [4], and an organization’s readiness for change has been defined as “a shared psychological state in which organizational members feel committed to implementing an organizational change and confident in their collective abilities to do so” [5]. The supra-individual level, i.e., the collective level above the individuals in the organizations, is important to study as collective behavior change is often required to implement multiple changes, such as in staffing, workflow, and organizational structures [5].

*Commitment* to change can be defined as the mindset that binds an individual to the course of action deemed necessary for the successful implementation of a change initiative [6]. Weiner proposes that organizational members whose commitment to change is based on “want to” rather than “have to” or “ought to” display not only more cooperative behavior (e.g., volunteering for problem-solving teams), but also championing behavior (e.g., promoting the value of the change to others) [5, 7]. Change *efficacy* refers to organization members’ shared belief in their “collective capabilities to organize and execute the courses of action involved in change implementation” [8]. It reflects the amount of knowledge available about what to do and how to do it, i.e., it is a function of members’ cognitive appraisal of three factors of implementation capability: the resources (including time) available, task demands, and the situation the organization faces [4, 9]. The ability to measure how an organization’s members perceive these two constructs may, ultimately, be used by health care leaders to develop more effective and efficient change strategies [4].

Like many countries, Denmark has begun a major system-wide structural reform to improve efficiency and the integration and coordination of health care [10]. The regions, hospitals, and clinical departments need to make considerable changes in service delivery, which requires the coordinated and collective actions of multiple organizational members to succeed. As part of a case study (not reported in this Short Report) to explore how a clinical department has managed this challenge [11], we wanted to use a validated instrument to test the organization’s readiness for implementing change.

As we could not find a Danish instrument, we chose the “Organizational Readiness for Implementing Change” (ORIC) questionnaire developed by Shea et al. as it is a promising, reliable, and valid instrument designed for health care, even though factor analysis has revealed some limitations and it has not yet been validated in an actual hospital setting [4]. In addition to undergoing a thorough psychometric assessment, ORIC is unique in comparison to other instruments [3] as it is grounded in theory, targets the supra-individual level, and its brevity suits the busy health care context [4]. Thus, a Danish translation of this instrument could both become a helpful instrument for Danish health care managers aiming to tailor implementation strategies in different health care settings [12]. In this Short Report, we assess the reliability and validity of a Danish version of the ORIC instrument in a hospital setting.

## Methods

### Participants and procedures

This study was conducted at the Department of Obstetrics and Gynecology (OB/GYN) at Aarhus University Hospital (AUH). To improve efficiency, integration, and coordination of health care services as required by the national reform, AUH started a hospital merger, downsizing of beds, and construction of a new hospital. Hospital management decided that the OB/GYN department should reduce beds by 36% and their budget by 10%, mainly by reducing nursing staff. The efficacy requirements were addressed through an improvement process that led to the implementation of changes at the level of care pathways for individual medical conditions and at the organizational level.

Organizational members were identified through employee lists provided by department managers. We crosschecked the list with department mailing lists to ensure all employees had been identified. We administrated the questionnaire electronically via SurveyXact (Aarhus/Denmark) and distributed it via email to all staff and managers in the OB/GYN department (*n* = 403) in June 2014. Informed consent was obtained electronically; only participants who gave their consent were able to answer the questionnaire. Reminders were sent out through September. The response rate was 72%. Key characteristics of the respondents are presented in Table 1.

**Table 1:** Baseline characteristics of the study participants, no. (%) unless otherwise indicated (*n* = 284)

Variable	No. (%)
Gender, female	264 (93.0)
Profession	
Nurse and Licensed Practical Nurse	109 (38.4)
Midwife	95 (33.5)
Physician	51 (18.0)
Medical secretary	21 (7.4)
Others	8 (2.8)
Age, years, mean (SD)	45, 9 (10.6)
Manager, yes	15 (5.3)
Permanent employed, yes	239 (84.2)
Length of employment, median (IQR)	18 (8.3, 27.4)

*SD* standard deviation, *IQR* interquartile range

### Measures

The “Organizational Readiness for Implementing Change” (ORIC) questionnaire contains 12 items that correspond to the domains of commitment and efficacy. It uses a 5-point Likert scale (1 = strongly disagree and 5 = strongly agree). To facilitate analysis, we grouped and labeled the items according to the domain they addressed (Table 2). The numbering (E1-E7 and C1-C5) corresponds to the additional file in the Shea article [4].

**Table 2:** The original English version of the ORIC (12 items) as presented in [4]. The Danish translation can be found in the supplementary file

Item number	Item description
Change efficacy (7 items)	
E1	People who work here feel confident that the organization can get people invested in implementing this change
E2	People who work here feel confident that they can keep track of progress in implementing this change
E3	People who work here feel confident that the organization can support people as they adjust to this change
E4	People who work here feel confident that they can keep the momentum going in implementing this change
E5	People who work here feel confident that they can handle the challenges that might arise in implementing this change.
E6	People who work here feel confident that they can coordinate tasks so that implementation goes smoothly
E7	People who work here feel confident that they can manage the politics of implementing this change
Change commitment (5 items)	
C1	People who work here are committed to implementing this change.
C2	People who work here will do whatever it takes to implement this change
C3	People who work here want to implement this change
C4	People who work here are determined to implement this change
C5	People who work here are motivated to implement this change

### Translation

The ORIC questionnaire was translated through the standard approach of “forward” and “back” translation [13, 14]. Forward translation was done by a native Danish speaker. Back-translation was done by an independent bilingual (English/Danish) native English speaker. The translations (see Additional file 1) were compared, discussed, and then refined. As recommended by Weiner, [5] the questionnaire was modified through the inclusion of an introductory description of the organizational change and to specific item sets so that it was clear what was meant by the phrase “this change”.

### Statistical analysis

Face and content validity of the translated questionnaire was assessed by Danish researchers working with organizational psychology and behavior research and who had conducted numerous studies within health care in Denmark and abroad.

Statistical analyses included Cronbach’s alpha to examine the reliability of the instrument, exploratory factor analysis (EFA) using principal axis factor analysis to evaluate dimensionality followed by a confirmatory factor analysis (CFA) to investigate constructs validity. We hypothesized that the two-factor representation efficacy and commitment of Weiner [4] would be replicated in this study.

Principal-axis factor analysis (FA) was chosen, because change commitment and change efficacy are interrelated, although independent, facets of organizational readiness [4, 5].

The number of factors to retain was determined using parallel analysis and eigenvalue-greater-than-one decision rule [15]. Following the principal axis FA, a CFA was performed to validate the resulting constructs from the EFA. The use of CFA to investigate the construct validity of hypothesis-based testing instruments adds a level of statistical precision. Maximum Likelihood Estimation was used to fit the CFA model. A full dataset was used with no missing value. Our sample size of 284 met the criteria of Myers et al. that includes as follows: *N* ≥ 200, ratio of *N* to the number of variables in a model (*p*), *N*/*p* ≥ 10 [16].

We employed the commonly reported indexes [14, 17, 18] to assess the fitness of the model: chi-square/df, CFI and root mean square error of approximation (RMSEA). The following cut-off values were used as the level of acceptance: CFI equal to or greater than 0.90, [19] RMSEA equal to or less than 0.08 [20], and CMIN/DF < 3. [17, 21] We considered Cronbach’s alpha values of 0.7 as acceptable for internal consistency. The level of significance was specified at 0.05.

Following the classical item analysis, Andrich’s extension of the Rasch measurement model for rating scale data was used [22] to evaluate the measurement properties of the ORIC. Rasch is often considered to be an item response theory (IRT) model. IRT is an important method of assessing the validity of measurement scales that is still underutilized [23]. It describes the properties of the items in the scale, and respondents’ answers to the individual items, item and person parameters, model fit statistics, and differential item functioning (DIF). The fit of response categories, items, and persons to the expectations of the model is evaluated by quality control fit statistics. These are infit mean square error and outfit mean square error [24]. For response categories, items, and persons, a fit (infit and outfit) value of 1.0 is perfect fit to the Rasch model. Fit values less than 1.0 indicate less variation in responses than the model expected while fit values greater than 1.0 indicate greater variability in responses than expected. The fit values for items should be between 0.5 and 1.5 for an item to have good fit to the model [24]. Rasch analysis computes two reliability coefficients for the measurement of person separation statistics—and model and real reliability to estimate the overall reliability of the scale. Reliability value larger than 80% associated with separation index greater than 2 is acceptable. Winsteps 4.0 was used for the Rasch model analyses [24]. SPSS 24 and Amos 24 were used for all other analyses (SPSS, IBM, Chicago). The level of significance was specified at 0.05.

## Results

Face and content validity was confirmed for all items with the exception of one. This referred to the word “commitment”, which is difficult to translate, as there is no Danish word that fully captures the English meaning used in item C1. Furthermore, there were minor comments regarding translations, such as the word “people” in all the items, which in the translation referred to the employees in the department, who were exposed to the change implementation.

The overall Cronbach’s alpha was 0.88. Efficacy is a seven-item factor (Cronbach’s *α* = 0.87) and commitment a five-item factor (Cronbach’s *α* = 0.75). Two factors were produced from the analysis: commitment and efficacy.

EFA results for the ORIC scale found two factors with an eigenvalue > 1. The screen plot also indicated the two factors. Most items loaded onto their respective factors (not presented). Items loaded on efficacy ranged between 0.54 and 0.76 and on commitment between 0.66 and 0.70.

CFA examined five models to evaluate the best fit for the overall data (Table 3). All items displayed statistically significant factor loadings on their respective factors. Model 1 includes all the items without any co-variance. Most of the loadings exhibited values between 0.62 and 0.86 for the factor efficacy (Table 4). The CFA results of model 1 showed the following values: CFI = .838, RMSEA = .113, and CMIN/DF = 4.796. For model 1, the chi-square test was significant (*p* < 0.001) and the other criteria for model fit were not met. The main difficulty presented in model 1 was that items E1 (Efficacy 1) and C1 (Commitment 1) had low standardized regression weights and the model did not fit as reflected by the fit indices. These items showed low factor loadings in both the EFA and CFA to warrant consideration of exclusion in the CFA.

**Table 3:** Results of the CFA by model and indices

Model	Absolute fit (RMSEA)	Incremental fit (CFI)	Parsimonious fit (Chisq/df)
Model 1	.113	.838	4.796
Model 2	.117	.841	5.000
Model 3	.067	.950	2.320
Model 4	.074	.943	2.603
Model 5	.071	.953	2.476

Model 1*—*all items without co-variance of the two factors; model 2*—*E1 is removed; model 3*—*E1 removed and the two factors were allowed to correlate; model 4*—*C1 removed and the two factors were allowed to correlate; model 5*—*C1 and E1 removed and the two factors were allowed to correlate

*RMSEA* root mean square error of approximation, *CFI* confirmatory fit index, *Chisq/df* chi-square/degrees of freedom

**Table 4:** Standardized regression weights of the items from the confirmatory factor analysis (CFA)

Items	Model 1	Model 2	Model 3	Model 5
E1	.644	–	–	–
E2	.724	.719	.712	.713
E3	.763	.740	.730	.729
E4	.729	.731	.738	.740
E5	.635	.661	.666	.669
E6	.721	.743	.740	.742
E7	.667	.665	.669	.670
C1	.402	.402	.416	–
C2	.461	.461	.488	.488
C3	.692	.692	.662	.673
C4	.707	.707	.716	.728
C5	.789	.789	.782	.777

*E* efficacy, *C* commitment

The results showed that model 3 has acceptable model fit with CFI = .95, RMSEA = .067, and CMIN/DF = 2.32 (Table 3). However, chi-square value for the overall model fit was significant (*p* < .001) suggesting a lack of fit between the hypothesized model and the data. We ignored this due to the sensitivity of chi-square in large samples as the sample size for study is greater than 200 [18, 25]. The assumption of normality was fulfilled.

A two factor (commitment and efficacy), 11-item scale, of the Danish language ORIC was shown to be valid. The constructs produced in the a priori model coincide with the constructs contextually available in the original article [4].

The Rasch analysis shows that the scale has acceptable person and item separation reliabilities but the assumption of unidimensionality was not satisfied, indicating that more than one latent construct is measured by a set of 12 ORIC items. The unidimensionality test is a Winsteps principal components analysis (PCA) of the standardized response residuals (the responses that are misfitting). The eigenvalue of the first contrast must be > 2.0 to suggest that a second dimension exists within the misfitting responses. The eigenvalue of the first contrast is 2.22. The three items with the strongest positive loadings on the first contrast were item C3 (.68), C4 (.67), and C5 (.57). The two items with the strongest negative loadings on the first contrast were item E3 (− .58) and item E2 (− .47).

All items have acceptable fit (Table 5). To evaluate if the Danish item calibrations differ by subgroups, a Mantel-Hanzel DIF analysis was conducted. No items with significantly different difficulty calibrations in age group, sex, management role, and profession were identified.

**Table 5:** Item statistics of the Danish version of ORIC

Item	Difficulty	Standard error	Infit	Outfit
C2	– .66	.08	1.47	1.43
C3	– .46	.08	1.39	1.39
C1	– 1.90	.10	1.25	1.23
E1	– .03	.08	.98	1.00
E5	.20	.08	.97	.95
C4	– .75	.09	.96	.96
C5	– .39	.08	.93	.89
E7	.69	.08	.88	.89
E6	.80	.08	.88	.86
E3	1.08	.08	.87	.87
E2	.93	.08	.81	.80
E4	.49	.08	.76	.75

For the Danish ORIC model, reliability is .86 and real reliability is .99 with a separation index of 2.47 and 9.69 respectively. The true reliability of the Danish ORIC model is somewhere between these two values. Reliability value larger than 80% associated with separation index greater than 2 is acceptable [24].

## Discussion

With a response rate of 72%, an 11-item scale of a Danish translation of the ORIC was shown to be valid and reliable when tested in a hospital setting. All items had acceptable fit in the IRT analysis and we did not observe significant DIF in subgroups of age group, sex, management role, or profession.

Corresponding to the theory and previous psychometric assessment [4, 5], factor analysis of the instrument demonstrated two correlated factors. However, the metrics in our study were slightly different. By removing Item E1 and allowing the factors to correlate, we identified a good fit of an 11-item model with a high factor loading. Shea et al. [4] obtained a good fit model by excluding two efficacy items.

Our ability to construct a model with a better fit may be due to the fact that our study was conducted in a hospital department facing a real organizational change effort. Thus, our study among hospital employees adds to Shea et al.’s study that validated the instrument among graduate and undergraduate students and NGO’s staff [4]. Like in the original article [4], item E1 (People who work here feel confident that the organization can get people invested in implementing this change) was excluded as it cross loaded on the commitment factor. The key phrase in item E1 is “invested in the change”. In Danish, the word invested was replaced with engaged because the phrase “investment” is in Danish exclusively associated with money, which may have contributed to the cross loading.

Recent studies using the English-language ORIC either had a small sample or surveyed only one representative of each workplace studied [26, 27], which challenge Weiner’s conceptualization of a collective measurement [5]. In both studies, the finding partly indicated that individual levels of commitment or efficacy can have been captured instead of the overall organizational levels, even though both studies used the word “We” in their questionnaire [26, 27]. In our study, we used a translation of the phrase “People who work here”, in accordance with the original version instead of “We”. We have not explored the implications of this difference. Another limitation is that we surveyed one clinical department, which may reduce generalizability. Future studies should focus on comparing different settings and determine the predictive value of ORIC through the use of organizational measures for instance on change efforts and performance outcomes. A further limitation is that we did not perform a concurrent validity assessment, to compare the ORIC results with other readiness measures.

A strength of this study is the use of both classical test theory and IRT. IRT offers a number of advantages by modeling the relationship of individual items to the construct measured. It provides a richer description of the performance of each item and greater detail on a measure’s precision compared to classical test theory, where a single estimate, such as Cronbach’s *α*, describes a measure’s reliability [23]. Thus, our study supports the English version as reliable and valid for health care settings. In the Danish context, the translated instrument can be used to further investigate the strategies hospitals employ to manage the current system-wide reform and the related changes in service delivery.

## Conclusion

The Danish version of ORIC showed acceptable reliability and validity as an instrument in a Danish-speaking population. It also confirms the validity and reliability of the ORIC instrument for hospital settings. Its brevity and theoretical underpinnings could make it an appealing and feasible tool for health care managers interested in evaluating their organizations and to tailor change strategies that better match staff views of the organization’s readiness for implementing change.

## Additional file

Additional file 1: Organizational Readiness for Implementing Change (ORIC)—Danish version. (DOC 46 kb)

## Abbreviations

SPSS, IBM: Statistical Package for the Social Sciences, International Business Machines Corporation
AUH: Aarhus University Hospital
CFA: Confirmatory factor analysis
CFI: Confirmatory fit index
DIF: Differential item functioning
EFA: Exploratory factor analysis
FA: Factor analysis
IRT: Item response theory
OB/GYN: Obstetrics and gynecology
ORIC: Organizational Readiness for Implementing Change
PA: Parallel analysis
RMSEA: Root mean square error of approximation
