# Supplementary material for: Assessing the reliability and validity of the Danish version of Organizational Readiness for Implementing Change (ORIC)
Source: Implement Sci. 2018 Jun 5;13:78. doi: 10.1186/s13012-018-0769-y (PMC5989337; doi:10.1186/s13012-018-0769-y)
Supplement: Supplementary file 1 — Organizational Readiness for Implementing Change (ORIC)—Danish version. (DOC 46 kb) [file 13012_2018_769_MOESM1_ESM.doc]

# Additional file 1 Organizational Readiness for Implementing Change (ORIC) – Danish version

| 1 | 2 | 3 | 4 | 5 |
| --- | --- | --- | --- | --- |
| Uenig | Delvis uenig | Hverken enig eller uenig | Delvis enig | Enig |

| 1. Personer, som arbejder her, føler sig sikre på, at organisationen kan få de ansatte engageret i gennemførslen af denne forandring. | 1 2 3 4 5 |
| --- | --- |
| 1. Personer, som arbejder her, arbejder på at gennemføre denne forandring. | 1 2 3 4 5 |
| 1. Personer, som arbejder her, føler sig sikre på, at de kan holde styr på fremgang i gennemførslen af denne forandring. | 1 2 3 4 5 |
| 1. Personer, som arbejder her, vil gøre hvad end der skal til for at gennemføre denne forandring. | 1 2 3 4 5 |
| 1. Personer, som arbejder her, føler sig sikre på, at organisationen kan understøtte de ansatte, imens de omstiller sig til denne forandring. | 1 2 3 4 5 |
| 1. Personer, som arbejder her, vil gerne gennemføre denne forandring. | 1 2 3 4 5 |
| 1. Personer, som arbejder her, føler sig sikre på, at de kan bibeholde drivkraft i gennemførselen af denne forandring. | 1 2 3 4 5 |
| 1. Personer, som arbejder her, føler sig sikre på, at de kan håndtere de udfordringer, der kan opstå under gennemførselen af denne forandring. | 1 2 3 4 5 |
| 1. Personer, som arbejder her, er fast besluttet på at gennemføre denne forandring. | 1 2 3 4 5 |
| 1. Personer, som arbejder, her føler sig sikre på, at de kan koordinere opgaver, så gennemførslen af forandringen går glat. | 1 2 3 4 5 |
| 1. Personer, som arbejder her, er motiverede for at gennemføre denne forandring. | 1 2 3 4 5 |
| 1. Personer, som arbejder her, føler sig sikre på, at de kan håndtere   politikken (magtstrukturer og evt. konflikter) omkring at gennemføre denne forandring | 1 2 3 4 5 |
